# Hierarchical Honeycomb-Structured Electret/Triboelectric Nanogenerator for Biomechanical and Morphing Wing Energy Harvesting

**DOI:** 10.1007/s40820-021-00644-0

**Published:** 2021-05-10

**Authors:** Kai Tao, Zhensheng Chen, Haiping Yi, Ruirong Zhang, Qiang Shen, Jin Wu, Lihua Tang, Kangqi Fan, Yongqing Fu, Jianmin Miao, Weizheng Yuan

**Affiliations:** 1 Ministry of Education Key Laboratory of Micro and Nano Systems for Aerospace, Northwestern Polytechnical University, X’ian, 710072 People’s Republic of China; 2 State Key Laboratory of Optoelectronic Materials and Technologies, Key Laboratory of Display Material and Technology, School of Electronics and Information Technology, Sun Yat-Sen University, Guangzhou, 510275 People’s Republic of China; 3 Department of Mechanical Engineering, University of Auckland, 20 Symonds Street, Auckland, 1010 New Zealand; 4 School of Mechano-Electronic Engineering, Xidian University, X’ian, 710071 People’s Republic of China; 5 Faculty of Engineering and Environment, Northumbria University, Newcastle upon Tyne, NE1 8ST UK; 6 School of Electronic Information and Electrical Engineering, Shanghai Jiao Tong University, Shanghai, 200240 People’s Republic of China

**Keywords:** Honeycomb-inspired structure, Morphing wing energy harvesting, Electret power generation, Triboelectric nanogenerator, Self-powered insole pressure mapping

## Abstract

Flexible, compact, lightweight and sustainable power sources are indispensable for modern wearable and personal electronics and small-unmanned aerial vehicles (UAVs). Hierarchical honeycomb has the unique merits of compact mesostructures, excellent energy absorption properties and considerable weight to strength ratios. Herein, a honeycomb-inspired triboelectric nanogenerator (h-TENG) is proposed for biomechanical and UAV morphing wing energy harvesting based on contact triboelectrification wavy surface of cellular honeycomb structure. The wavy surface comprises a multilayered thin film structure (combining polyethylene terephthalate, silver nanowires and fluorinated ethylene propylene) fabricated through high-temperature thermoplastic molding and wafer-level bonding process. With superior synchronization of large amounts of energy generation units with honeycomb cells, the manufactured h-TENG prototype produces the maximum instantaneous open-circuit voltage, short-circuit current and output power of 1207 V, 68.5 μA and 12.4 mW, respectively, corresponding to a remarkable peak power density of 0.275 mW cm^−3^ (or 2.48 mW g^−1^) under hand pressing excitations. Attributed to the excellent elastic property of self-rebounding honeycomb structure, the flexible and transparent h-TENG can be easily pressed, bent and integrated into shoes for real-time insole plantar pressure mapping. The lightweight and compact h-TENG is further installed into a morphing wing of small UAVs for efficiently converting the flapping energy of ailerons into electricity for the first time. This research demonstrates this new conceptualizing single h-TENG device's versatility and viability for broad-range real-world application scenarios.

**Supplementary Information:**

The online version contains supplementary material available at 10.1007/s40820-021-00644-0.

## Highlights

- Create a hierarchical honeycomb-inspired triboelectric nanogenerator (TENG) with excellent transparency, compactness, lightweight and deformability.
- Amplify capacitance variation by dividing large hollow space into numerous energy generation units with porous honeycomb architecture.
- Demonstrate self-powered insole plantar pressure mapping applications by the self-sustained elastic nature of the h-TENG device.
- Integrate the h-TENG into the morphing wing of small-unmanned aerial vehicles for converting flapping motions into electricity for the first time.

**Supplementary Information:**

The online version contains supplementary material available at 10.1007/s40820-021-00644-0.

## Graphical abstract

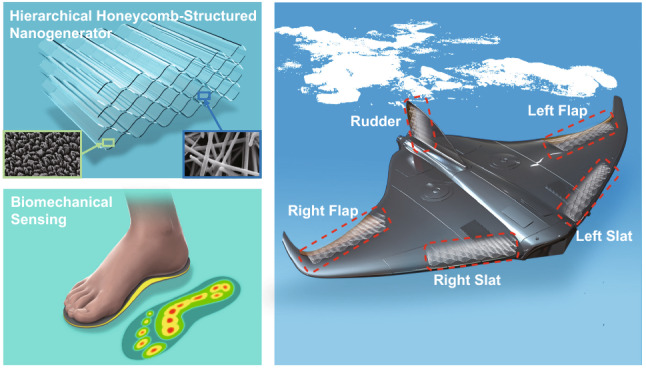

## Introduction

Recent advances in Internet of Things (IoT) have witnessed the rapid development of intelligent sensor nodes with characteristics of low power consumption, flexibility and sustainability [1–3]. Conventional power supply technologies with electrochemical batteries or power adapters will not be the preferred choice due to their limited lifespan, complicated maintenance and environmental unfriendliness. Energy harvesting systems as self-sustained power sources are capable of harvesting and transforming unused ambient energy into electricity. Such energy harvesters provide alternatives to those conventional electrochemical batteries and could pave an important step for the realization of self-autonomous operation and intelligent sensing [4–10].

There are four major transduction mechanisms for converting mechanical energy into electrical energy, namely piezoelectric [11–13], electromagnetic/magnetostrictive [14–19], electrostatic [20–23] and triboelectric [24–30]. Triboelectric nanogenerators (TENGs) were first proposed by Professor Zhonglin Wang’s group in 2012. It is based on the coupling of contact electrification and electrostatic induction, where electron migration is created when two materials with dissimilar electron attraction are contacted. Redistribution of electrons takes place when two friction materials contact/separate each other under mechanical excitations. Electromagnetic/magnetostrictive transduction mechanism is based on Faraday’s law of induction with changing of magnetic flux in a closed coil, either by relative movement (electromagnetic) or mechanical strain (magnetostrictive). The electrostatic mechanism is based on variable capacitor structures with a constant voltage/charge bias. The bias can be provided either by pre-charged electrets or by an external power source. Piezoelectric harvesters usually operate on stretching or compressing piezoelectric materials, such as piezoelectric polymers and piezoelectric ceramics, thus transforming mechanical strain or stress into electricity due to the piezoelectric effect [31–35]. Among these mechanisms, the triboelectric nanogenerator presents numerous advantages in terms of high output voltage and efficiency, ease of structural design and fabrication, diverse material selection, low-cost and broad low-frequency application scenarios such as human motion and aircraft morphing wing oscillation [25, 26, 36–38].

To improve the performance of TENGs, numerous efforts have been made in various aspects, including multilayer-stacked structures [39–52], nanostructured surface modifications [53–55], contact material synthesis [56, 57], optimization of surface charge density [58–60], advanced mechanical design [61–65], novel manufacture approaches and power management circuit design [66–68]. Among these, multilayer-stacked TENG has been regarded as one of the most effective and simple methods to maximize the instantaneous output power by synchronizing all the layers of TENGs. Both contact triboelectrification and electrostatic induction can be significantly enhanced by multiplying contact area variations and capacitance variation within a confined space. Table S1 summarizes the state-of-the-art TENGs with multilayered structures for improving the performance of TENGs in various aspects, including substrate material, device volume, weight and area, primary structure, connected method, etc. However, these approaches still encounter some significant challenges:

- To sustain periodic contacts and separation operations, most multilayered or arch-shaped TENGs require auxiliary architectures to provide elastic support, making the whole device rigid, heavy-weight and bulky. Therefore, they may have difficulties in integrating with wearable or biomechanical microsystems [45, 46].
- The charge circulation of TENG is mainly driven by capacitance variation of electrostatic induction. The maximum power generation occurs only when the two electrodes are at a very close position, where the capacitance variation reaches its maximum value. This indicates that the power generation efficiency per unit volume is not high if the initial stage's air gap is too large. Therefore, a small air gap in the initial stage is often required for high output performance and high-power density [39, 48, 69].
- The output power of TENGs is quadratically proportional to the triboelectric surface charge density. The electrostatic induction of TENGs is severely affected by the limited surface charge density, which only relies on contact triboelectrification. Therefore, the charge trap capability is not fully exploited, except contact triboelectrification processes [58].
- Current TENGs with rigid and burdensome architectures usually restrict their operation modes, making it non-adaptable to excitation vibrations with irregular, random and low-frequency characteristics, such as morphing wing motions.

**Fig. 1:**
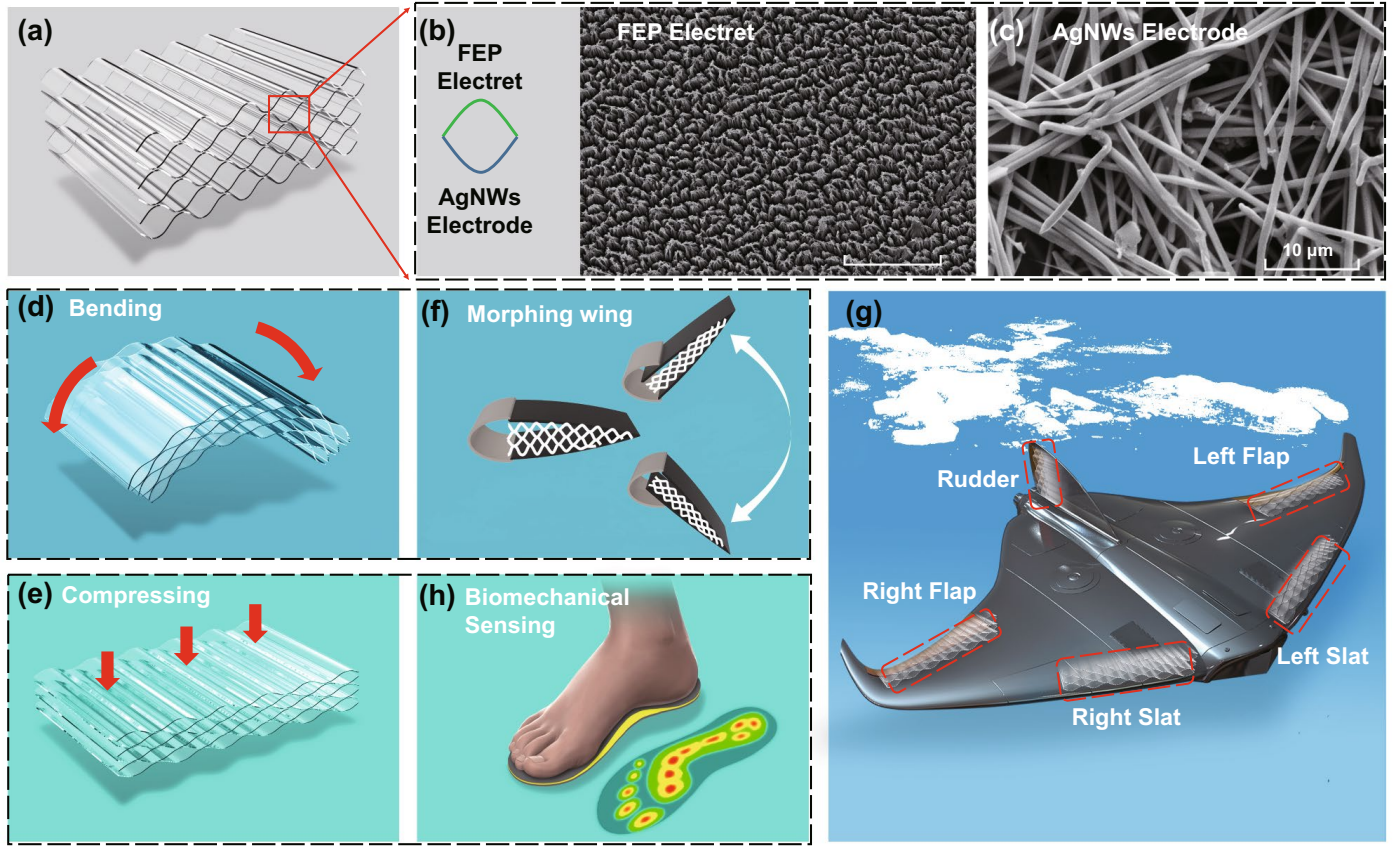
**a** Schematic structure of the proposed hierarchical honeycomb-structured electret/triboelectric nanogenerator. **b**, **c** Architecture of single arch-shaped deformable energy generation unit within honeycomb cells: SEM images of the FEP thin film with a nanostructured surface (top layer) and surface morphology of the fabricated AgNWs electrode film (bottom layer). **d**, **e** The proposed h-TENG operates at bending mode and compression mode. **f**, **g** A schematic illustration of the power generation mechanism of the morphing wing by replacing the honeycomb cellular mesostructure with the lightweight and compact h-TENGs skin. **h** The proposed h-TENGs can be integrated into the insole for real-time plantar pressure mapping applications

Herein, we propose a honeycomb-inspired TENG with multilayer-wavy-structured fluorinated ethylene propylene (FEP) electret thin films to address the above issues (Fig. 1a). Nanostructured pillars were created on the FEP thin film surface by an ion beam etching process to increase the contact area during triboelectrification (Fig. 1b). Transparent polyethylene terephthalate (PET) film synthesized with conductive silver nanowires (AgNWs) is employed as a flexible substrate to sustain cycling pressing and releasing operations. The scanning electron microscope (SEM) image of the AgNWs electrode film's surface morphology shows that the AgNWs film surface roughness is within a range of ~ 82 to 78 nm (Fig. 1c). Such a high surface roughness and many defects between AgNWs and FEP electret are beneficial for capturing more electrical charges, which are beneficial for the performance of the h-TENGs. Furthermore, this newly proposed h-TENG device exhibits several unique advantages:

- Stacked multilayer h-TENGs can be conveniently constructed through high-temperature thermoplastic molding and wafer-level bonding of multilayer FEP/PET/FEP composites to form honeycomb structures. These facile and low-cost formation processes are applicable for future manufacture with a mass production capability.
- The elastic nature of honeycomb cellular mesostructures and PET/AgNWs substrate endows the h-TENG device with excellent resilient and self-rebounding properties. Therefore, no auxiliary resilient supports are required, making the whole device compact, flexible, transparent and lightweight.
- The porous honeycomb architecture has divided the sizeable hollow space into numerous energy generation cells. The overall capacitance variations can be significantly amplified by synergizing all the energy generation cells with small air gaps, making the proposed h-TENG extremely sensitive to external perturbations.
- The honeycomb-structured TENG proposed in this research adopts a novel 3-D hierarchical structure based on multilayer PET/AgNWs/FEP composites compared with the previous TENGs with in-plane honeycomb patterns [70–72]. These parallel-connected and deformable units can significantly boost capacitance variations and enhance the overall performance of the device. Both the coupling of contact electrification and electrostatic induction can be multiple improved within a confined space.
- Corona discharging process is further utilized to maximize the surface charge density of the thin film electret. Therefore, high energy output can be readily obtained by full use of electret thin films' charge trap capability.

The fabricated h-TENG has such properties of ultra-lightweight, long-term durability and excellent flexibility. Therefore this newly proposed device can be integrated into unmanned aerial vehicles (UAVs) for harvesting kinetic energy from morphing wing motions. Figure 1d, e show the two operation modes of the proposed h-TENG: bending mode and compression mode. Figure 1f, g show schematic illustrations of the morphing wing's power generation mechanism by replacing the honeycomb cellular mesostructure within UAVs with the proposed lightweight and compact h-TENGs skin. The h-TENG device can be further integrated into the insole for real-time plantar pressure mapping applications (Fig. 1h). An instantaneous open-circuit voltage of 1207 V and a short-circuit current of 68.5 μA are generated by gently tapping the h-TENG by hand. As a real-time self-powered tactile sensor, the 5 × 5 h-TENG array is capable of lighting up a 10 × 10 LED display panel for instantaneous pressure mapping, which will be deliberated in the subsequent sections.

## Experimental Sections

### Synthesis of AgNWs on PET Surface

PET thin film (Hefei Vigon Material Technology Co., Ltd) with a thickness of 30 μm is adopted as the substrate material. The PET thin film is firstly treated with alginate for 2 min and then brushed with AgNWs using a rolling/pressing process to achieve good conductivity. After solidifying AgNWs coated PET film at room temperature, CaCl_2_ and chlorine chloride are introduced to enhance the bonding of the AgNWs onto the surface of PET thin films. Finally, 120-nm-thick conductive AgNWs thin film is successfully synthesized on the surface of PET to form transparent PET/AgNWs composite, which is used as both the conductive electrode and elastic substrate for the h-TENG device.

### High-Temperature Molding of PET/AgNWs/FEP Composite

A vacuum hot-pressing process is applied to bond 50-µm-thick FEP electret thin film (Goodfellow Cambridge Co., Ltd) onto the surface of PET/AgNWs film to achieve a good contact and adhesion. The PET/AgNWs/FEP composite is then pressed together using a complementary wavy-shaped iron alloy mold and silica gel at a temperature of 80 °C and an applied force of 1 kN for 10 min. The complementary iron (Fe–Ni) alloy template with a size of 5 × 3 × 4 cm^3^ is used for the molding process. The silica gel is used to protect AgNWs, thus maintains a good conductibility during the hot pressing.

### Assembly of the h-TENG Device

The ionized charges are pre-implanted into the wavy-structured electret composites of PET/AgNWs/FEP through a corona discharging process under a − 4 kV voltage for 10 min. The wavy-structured PET/AgNWs/FEP composite is then turned over and positioned onto the flat surface of the ethoxyline-resin-coated wafer. Therefore, only the backside nodal line of the wavy structure is coated with ethoxylate resin. Finally, the multilayered honeycomb structures and h-TENG devices are fabricated through a layer-by-layer stacking process of PET/AgNWs/FEP composites.

### Measurement of the h-TENG Device

A high-impedance data acquisition system (DAQ, NI USB-6289 M series, USA, impedance $>$ 10 GΩ) was used in recording the electrical signal. The fabricated h-TENG was sandwiched with two parallel plates. The top plate is fixed to a 3D positioning stage, which controls the h-TENG prototype's height. The bottom plate is mounted on the top of the electromagnetic vibrator, which can provide sinusoidal vibration excitation at tunable amplitudes and frequencies. The capacitance of h-TENG is measured using a precision LCR meter (Applent AT811, CN). In the mechanical and electrical property characterization, the frequency of excitation acceleration and load resistance are set at 20 Hz and 500 MΩ, respectively.

## Fabrication and Working Principle

### Fabrication of h-TENG Device

**Fig. 2:**
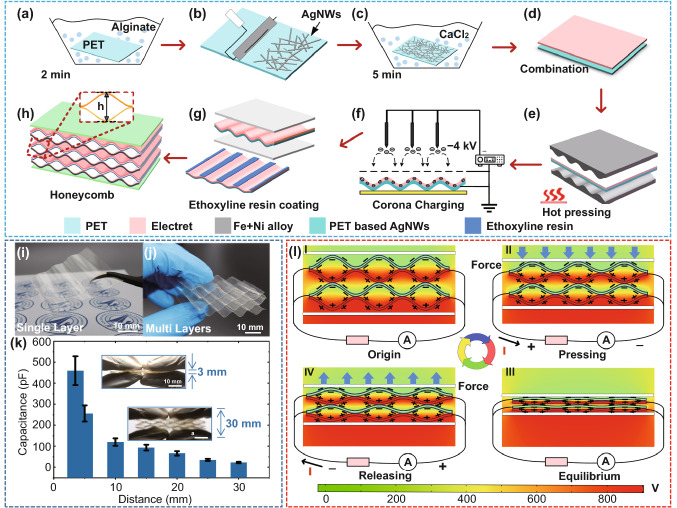
Fabrication processes and working mechanisms of the proposed h-TENG: **a** PET thin film is treated with alginate for 2 min. **b** AgNWs are pressed onto the surface of PET by rolling process. **c** CaCl_2_ treatment is used to ‘weld’ the AgNWs onto the surface of PET thin film. **d** FEP electret thin film is bonded onto PET/AgNWs film surface with ethoxylate resin. **e** A single-wavy structure is formed using iron alloy templates through high-temperature molding process. **f** A corona discharging process is used to implant charge into FEP electret thin films. **g** Honeycomb structures are assembled by stacking multilayered PET/AgNWs/FEP composite together. **h–j** schematic illustration and optical photographs of the fabricated h-TENG device. **k** Capacitance values change from 20 to 480 pF when the height of h-TENG changes from 3 cm to 3 mm with stacking triple-layer wavy structure. **l** Electric field and charge redistribution within four stages of h-TENG in a compress-release cycle: I Initial stage of original state. II Electrostatic induction by electrets when two electrodes get close to each other under external compressive force. III Contact triboelectrification when two electrodes get intimate contact with each other. IV Charge flows back when releasing

Figure 2a–h schematically illustrate the fabrication process of the h-TENG device. PET thin film is adopted as the substrate material since it has the excellent flexibility and transparency. The PET thin film is firstly treated with alginate (Fig. 2a) and then brushed with AgNWs (Fig. 2b). CaCl_2_ and chlorine chloride are introduced to enhance the adhesion between AgNWs and PET thin films (Fig. 2c). The Cl^−^ in the chlorine chloride peels off the stabilizer on the surface of the AgNWs and then induces a ‘welding effect’ of AgNWs, thus reduce the contact resistance. In the meanwhile, the Ca^2+^ in the CaCl_2_ reacts with alginate on the substrate to form an alginate gel, which improves the adhesion between the AgNWs and substrate. Therefore, transparent PET/AgNWs composite is successfully synthesized as the elastic substrate for the h-TENG device. Figure 2d shows wavy-structured PET/AgNWs/FEP composites are formed through a vacuum hot-pressing process since the PET thin film has excellent thermo-plasticity with a low Young's modulus of only 2.7 GPa. The period and amplitude of the wavy sheet can be controlled by adjusting the iron alloy template parameters, as shown in Fig. S1. The silica gel is used to protect AgNWs, thus maintains a good conductibility during the hot pressing.

The setup of vacuum hot-pressing machine and PET deformation properties under different temperatures are shown in Figs. S2a and S2b, respectively. To maintain a good conductivity of AgNWs/PET composites, the molding temperature should be adjusted below the glass transition temperature of PET to achieve a good stability of wavy structures while maintaining moderate stress. Figure S2c shows the stability characterization results of electret surface potentials and wavy amplitude variations of PET/AgNWs/FEP composites after continuous operation of ~ 10,000 cycles. Results show that a proper elasticity of PET and a reasonable charge stability of electrets can be achieved when the molding temperature is controlled at around 80 °C. This guarantees a good elasticity of the wavy-shaped PET, regardless of various forces applied during the assembly process.

The FEP thin film, applied as the electrets, can provide an electric field for years through quasi-permanent electric charges or dipole polarization generated after the discharging process (Fig. 2f). An ion beam etching process is applied to create a nanostructured surface of FEP thin film for increasing the contact area during triboelectrification. The corona discharging process can maximize the charge density of FEP electret thin films for improving the performances of both the contact electrification and non-contact electrostatic induction. Figure 2g–j show schematic illustration and optical photographs of the h-TENG device fabricated through a layer-by-layer stacking process of wavy PET/AgNWs/FEP composites. The green sheet in Fig. 2h is h-TENG substrate material, which is made by flexible printed circuit board (FPCB) technology. FPCB is utilized for wiring connection and integration of TENG arrays. Figure S3 shows that good transparency is still maintained after stacking several PET/AgNWs/FEP layers together. The electrical resistance of PET/AgNWs is examined using a four-probe electrical resistivity measuring technique, and results show that the wavy-shaped PET/AgNWs still maintains 95% conductivity of the planar PET/AgNWs sheet. The PET/AgNWs thin film's original square resistance is around 38 Ω sq^−1^ and increased to 40 Ω sq^−1^ after the vacuum hot-pressing process. The fabricated h-TENG is capable of integrating into wearable electronics for biomechanical energy harvesting and self-powered sensing applications.

### Working Mechanism

The porous honeycomb architecture has separated the h-TENG into a parallel-connected array of arch-shaped deformable capacitors. These parallel-connected and deformable energy generation units are beneficial for boosting the overall power output and increasing the whole h-TENG device's flexibility. For the electrostatic induction or triboelectrification, the maximum output power is generated when the two electrodes are at their closest positions. Theoretically, the amount of capacitance variation when the distance of two electrodes is changed from 1 mm to 1 µm is about ten times higher than when the corresponding distance is changed from 1 cm to 1 mm. It indicates that by dividing a large hollow structure into many small and parallel-connected capacitors, the overall performance of the h-TENG is enhanced significantly, especially when the displacement of the electrode is comparatively tiny. For example, results shown in Fig. 2k demonstrates that the capacitance value is increased from 20 to 480 pF when the height of the h-TENG is decreased from 30 to 3 mm with a stacked triple-layer wavy structure. In contrast, the capacitance is only increased from 8.2 to 76 pF for a single-layer system under the same condition. Therefore, due to these two strategies' synergistic effects, a high performance of energy harvesting can be readily obtained with the proposed h-TENG device.

Finite element method (FEM) via COMSOL Multiphysics software is employed to understand the electric field and potential redistribution during the compress-release cycle of the h-TENG device. Figure 2l presents a cross-section view of the geometrical layout and electric field distribution of a two-layer structured h-TENG device. In the initial state, the electric field is established between the FEP layer and the counter electrode. The net charges and dipoles that existed in the FEP form a constant bias surface potential when the counter electrode is grounded (Stage I). When the distance of two electrodes of the arch-shape energy conversion unit is decreased due to the applied external compress force, electrostatic induction is generated by the pre-implanted charges in the FEP electrets. It drives the induced charges flowing in the external circuit (Stage II). The amount of redistributed charges are kept increasing until the two electrodes of the arch-shaped energy conversion unit are intimately contacted with each other, when the triboelectrification effect occurs. At this stage, surplus charge is created due to different work functions of two dissimilar contact surfaces (Stage III). After the external force is removed, the arch-shaped energy conversion unit of the h-TENG recovers to its original shape due to the internal self-bounding ability of the PET material, which results in the induced charges flowing back to the counter electrode. Finally, when the generator is compressed again, a periodic current is generated by pressing and releasing the process intermittently (Stage IV). In this way, both the electret-based electrostatic induction and contact triboelectrification are engaged during the charge circulation process, leading to a superior charge synchronization within this unique honeycomb multilayered structure.

## Results and Discussion

### Mechanical and Electrical Property Characterization

Various mechanical and electrical parameters of the h-TENGs are investigated in order to optimize their output performance, including the initial wavelength, wave amplitude, honeycomb height and layer, external acceleration excitation and load resistance. Mechanical properties of the proposed h-TENG are highly dependent on the thermoplastic process of wavy-structured PET/AgNWs/FEP composites. Therefore, the wavelength and amplitude of wavy structures need to be optimized and controlled by iron alloy templates' mold parameters.

**Fig. 3:**
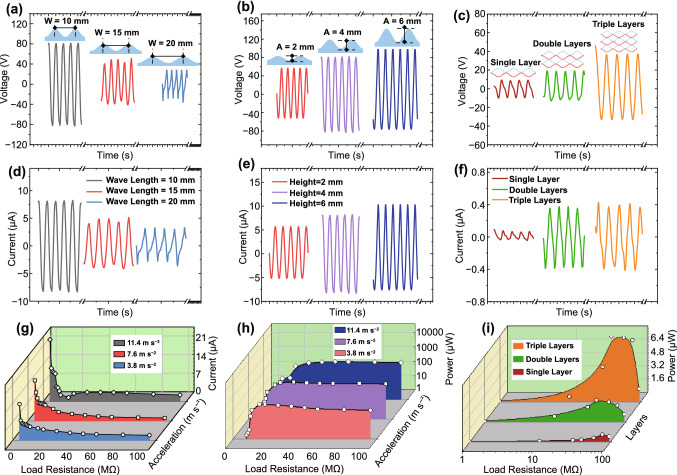
Optimizations of mechanical and electrical parameters for the h-TENG: **a** Output voltage waveforms of h-TENG at a constant wave amplitude of 4 mm and different wavelengths ranging from 10 to 20 mm. **b** Output voltage waveforms of h-TENG with a constant wave wavelength of 15 mm and different amplitudes of 2 mm, 4 mm, 6 mm. **c** Output voltage waveforms of h-TENGs with from a single-layer wavy thin films to triple-layer films. **d**–**f** The corresponding output currents of h-TENG with different wavelengths, amplitudes and layers of wavy PET/AgNWs/FEP composites. **g**, **h** Output current and power optimizations with varying accelerations of excitation and load resistances. **i** Output power optimizations with different layers and load resistances

Figure 3a shows the output voltage waveforms of the wavy h-TENGs with a constant wave amplitude of 4 mm and different wavelengths ranging from 10 to 20 mm. It can be seen that with the wavelength increased from 10 to 20 mm, and the output voltage is decreased from 80 to 35 V. The decrease of output voltage is mainly due to the slower capacitance variations and smaller contact surface area. Figure 3b shows the output voltage waveforms of h-TENG with a constant wave wavelength of 15 mm and different amplitudes of 2, 4 and 6 mm. It is observed that with higher wave amplitude, the output voltage is increased from 43 to 92 V under the same excitation. This is because a larger capacitance variation can be generated with a larger air gap. Therefore, the wavelength decrease and height increase are beneficial for improving the output performance of the h-TENGs. However, due to fabrication restrictions and conduction problems associated with substrates with larger curvatures, the rational values with a wavelength of 15 mm and an amplitude of 2 mm are adopted in the later design of the honeycomb structure. Figure 3c demonstrates that the output voltage is amplified by 2.7 times when the layers of wavy PET/AgNWs/FEP thin films are increased from one to three. It indicates that increasing the power generation layers is the most effective method to improve the performance of the h-TENG. Figure 3d–f show the corresponding output currents of h-TENGs with different wavelengths, amplitudes and layers of wavy PET/AgNWs/FEP composites, demonstrating the similar characteristics and trends with that of output voltages.

Parametric studies are further extended to optimize the performance of h-TENG under different load resistances, excitation accelerations and layers. The fabricated h-TENG has a wavelength of 15 mm, a height of 2 mm and a triple-layer structure. Figure 3g, h show 3D plots of data variations for electrical currents and output powers at different accelerations and load resistances. Results show that the short-circuit currents are increased from 13.6 to 24.2 µA with the increased excitations from 3.8 to 11.4 m s^−2^. The optimum powers of 266, 389 and 452 µW are obtained at excitation accelerations of 3.8, 7.6 and 11.4 m s^−2^, respectively. Figure 3i shows that the optimum output powers of 0.9, 2.6 and 7.8 µW are obtained at load resistances of 80, 40 and 35 MΩ with single, double and triple-layer structures, respectively. The maximum power is usually obtained at the smallest optimum load resistance with the increased number of power generation layer of the h-TENG. With the same prototype, a higher output power is normally associated with a larger capacitance variation, which can be obtained by increasing either excitation accelerations or power generation layers. The optimum impedance is reported to be reversely proportional to capacitance variation $R \propto \frac{1}{j\omega C}$ [73]. Therefore, a smaller optimum resistance of the proposed h-TENG is obtained with the increase of power generation layers.

### Power Generation with Human Motion

**Fig. 4:**
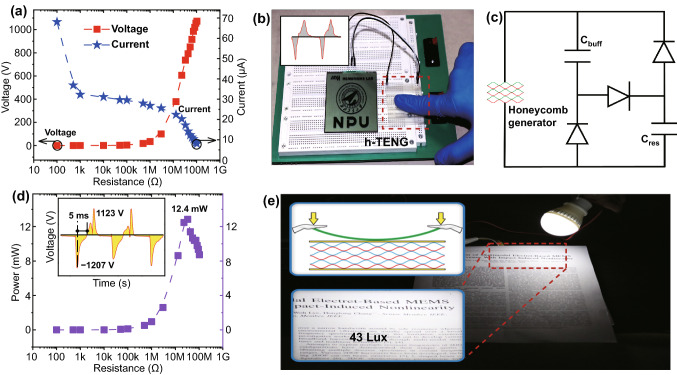
**a** Output voltages and currents of h-TENG with different load resistances by hand tapping. **b** Lighten up 75 × 55 mm^2^ LCD with h-TENG by finger touching. **c** Bennet’s doubler conditioning circuit for driving external electronics. **d** Output power optimization at different load resistances with hand tapping. **e** Light intensity of globe bulb up to 43 lx obtained by continuously tapping the h-TENG for ten times with a buckled ruler

The flexible and lightweight h-TENG is convenient for wearable or human motion energy harvesting applications. Figure 4a shows the derivations of output voltages and currents of a single-layer h-TENG device with different load resistances under the human palm's pressing excitation. It is seen that the short-circuit current and open-circuit voltage can reach up to 68 µA and 1123 V, respectively. By a simple finger pressing, the h-TENG can efficiently lighten up a 75 × 55 mm^2^ LCD with a logo of ‘NPU’ (Fig. 4b). To obtain a better visualization of power generation demonstration, we connect a Bennet’s doubler conditioning circuit for driving the external electronics (Fig. 4c). The Bennet’s circuit is capable of rectifying output AC signals from the h-TENG device to unidirectional DC signal, then effectively boosting the voltages and storing the energy into the capacitor. With the aid of Bennet’s circuit, the 75 × 55 mm^2^ LCD can be lightened up for 83 s by simply hand pressing the h-TENG device ten times, as shown in Video S1. After that, the LCD brightness is gradually reduced.

The output power optimization at different load resistances with simply hand tapping is shown in Fig. 4d. Triggered by gentle hand tapping, the fabricated h-TENG prototype produces instantaneous open-circuit voltage, short-circuit current and output power, with their maximum readings of up to 1207 V, 68.5 μA and 12.4 mW, respectively, corresponding to a remarkable peak power density of 0.275 mW cm^−3^ (or 2.48 mW g^−1^) using the hand tapping. Based on the inset Figure's output waveform shown in Fig. 4d, the restoring time of the h-TENG takes only ~ 5 ms. The excellent responsivity of the h-TENG is suitable for self-powered sensor for instantaneous sensing applications.

Figure 4e shows the photograph of a globe bulb that is powered by the h-TENG. The h-TENG device is squeezed for ten times with a buckled ruler. The generated power is firstly stored in a 47 μF capacitor through the Bennet’s circuit and then discharged to lighten up the globe bulb. The intensity of light is characterized using a digital lux meter (GM 1020). The results show that the light intensity can be increased up to 43 lx, which is able to use to lighten up a printing paper, as shown in the inset of Fig. 4e. Figure S4 in the supplementary information also demonstrates that the flexible h-TENG can be integrated into a smart foam mat, collecting kinetic energy from a person’s repeated stepping and jumping exercises.

### Self-powered Sensing Demonstration

**Fig. 5:**
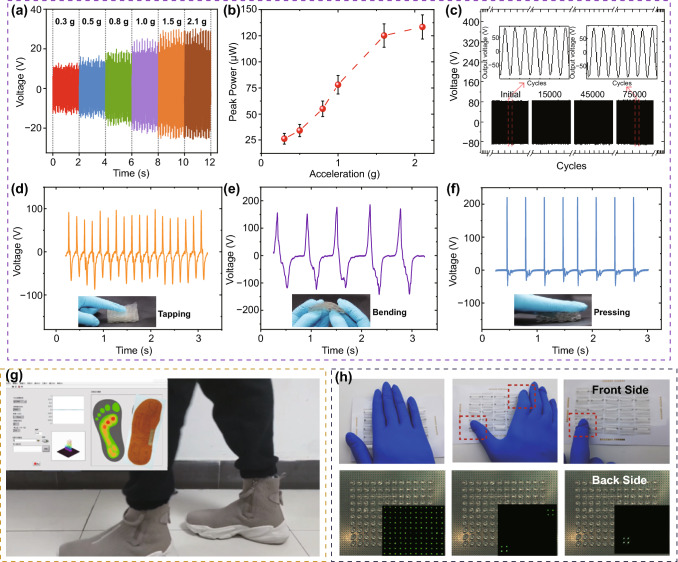
**a**, **b** Output voltage waveforms and magnitudes versus different excitation accelerations in the range of 0.3 ~ 2.1 g. **c** Output voltages of proposed h-TENG under continuous operations for ~ 75,000 cycles at an excitation acceleration of 5 g and a frequency of 30 Hz. **d**–**f** Output signal characteristics of proposed h-TENG by human motions, such as finger touching, bending and tapping. **g** Snapshot of real-time insole plantar pressure mapping using an array of h-TENGs during human walking. **h** Lighten up 10 × 10 LED display panel by 5 × 5 h-TENG array for instantaneous hand force mapping, when the whole palm, one finger or two fingers touch the generator array

The output characteristics of h-TENG are further studied with continuous periodic excitations under different accelerations. With an excitation frequency of 20 Hz and an external load resistance of 10 MΩ, the time-domain waveforms and output voltage magnitudes versus different excitation accelerations in the range of 0.3 ~ 2.1 g are shown in Fig. 5a, b, respectively. It is found that the output performance is in a quasi-linear relationship with the excitation accelerations below 1.6 g. It demonstrates the applicability of h-TENG for a self-powered accelerometer or force sensor in this acceleration range. After that, the stiffness of the h-TENG becomes significantly higher with enhanced rebounding ability and responsivity due to the decreased plasticity of the PET thin films.

To investigate the stability and long-term durability, the h-TENG device is further characterized by continuous operation for ~ 75,000 cycles at an excitation acceleration of 5 g and a frequency of 30 Hz, with the tapping motion generated using a mechanical shaker. According to the obtained results shown in Fig. 5c, there is no noticeable performance degradation of the h-TENG, reflecting its ultra-robustness and good stability after such a long-term operation. The excellent performance of the proposed h-TENG should be attributed to the excellent mechanical properties of honeycomb structures and the stable thermo-plasticity of PET/AgNWs/FEP composites.

Due to the excellent elastic property and self-rebounding honeycomb structure of the h-TENG, the flexible and transparent device can be easily pressed, bent and integrated into wearable devices. Figures 5d–f demonstrate output signals of the proposed h-TENG by human motions, such as finger touching, bending and tapping. Due to different frequencies and deformation characteristics, the output voltage signals demonstrate significant differences in their output voltage amplitudes, frequencies and waveforms. During bending experiments, two hands were used to hold the h-TENG to compress and bend the device to the degree of 90° and then release it at a slower frequency of 2 Hz to simulate the daily hand bending movements. The hand bending of h-TENG generates the lowest frequency but with the most symmetrical waveforms. While tapping, two fingers at the frequency of 6 Hz were applied on the h-TENG to simulate the state of the fabricated device being tapped during self-powered sensing applications. The finger tapping motion generates the lowest output voltages, whereas the hand pressing motion produces the highest output voltages but with the most asymmetrical waveforms. The experimental results indicate that different human motions can be distinguished using the proposed flexible h-TENG.

Insole plantar pressure mapping is critical for sport injury prevention, foot ulceration prediction and biomechanics information acquisition, etc. The above experiments have already demonstrated that the proposed h-TENG device has an excellent self-restoring ability and a fast response. Therefore, it has a great potential to be used for self-powered insole plantar pressure mapping and tactile imaging.

In order to integrate the h-TENG into the insole, the volume of each h-TENG unit is decreased to 20 × 15 × 10 mm^3^. The insole substrate is made from a flexible printed circuit board (FPCB) with its integrated circuit design and wire connection pads. Firstly, an array of ten h-TENG devices are bonded to the FPCB and wired to the connection pad onsite. The output signal is connected to a signal acquisition card (NI-USB6289). After that, the whole device was installed into the tester’s shoes. Secondly, the outputs of the multi-arched h-TENGs were acquired by a total of 10 integrated circuits on the signal acquisition card. The 20 analog input channels were real-time sequential scanned by the customized data real-time processing and graphics display procedure based on LabVIEW Software, which is able to normalize the output signal and visualize the output magnitudes with different colors. Thirdly, the output signals were transferred into the graphical data to display the walking tester's foot pressure distributions. Figure 5g shows an example of a snapshot of real-time insole plantar pressure mapping by using an array of h-TENGs during the walking of a person. The real-time video of insole plantar pressure mapping during the person’s walking is shown in Video S2. It can be clearly seen that the person is walking with these smart insoles. The pressure distribution of the insole can be real-time displayed on the computer screen with various color differences.

A square shape of 5 × 5 array of h-TENG devices is further integrated with a white FPCB board for tactile hand pressure mapping. On the back of the PCB, a 10 × 10 LED lamp array has been applied, and each h-TENG is linked to four series-connected LED lamps. The LEDs at different locations can be lightened up when the hand touches different positions of the device. Figure 5h demonstrates that the back LED has been lightened when the whole palm, one finger, or two fingers touch the generator array, respectively. It can be clearly observed that the LED lights can be lightened up when different hand excitations are applied to the various locations, reflecting the ability of the cellular h-TENGs for self-powered and real-time touch imaging applications.

### Aircraft Morphing Wing Energy Harvesting Demonstration

**Fig. 6:**
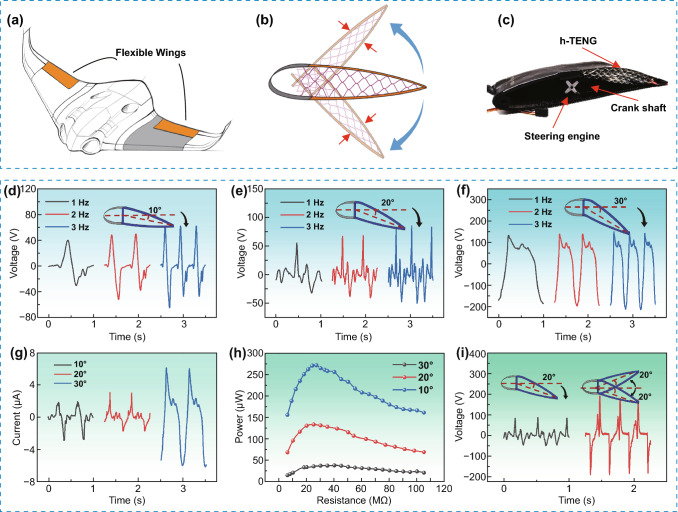
**a** Morphing wing of the unmanned aerial vehicle (UAV). **b** A schematic view of the cross-section of the morphing wing, where honeycomb cellular mesostructure is replaced by light and compact h-TENGs skin. **c** A digital photograph of the fabricated morphing wing, which is controlled by a motor through non-conformal contact for adjusting the flap angle. **d**–**f** Output voltage waveforms of h-TENG at various excitation frequencies under different wing deflected angles of 10°, 20°, 30°. **g** Output currents of h-TENG with different wing deflected angles at a frequency of 2 Hz. **h** Output power comparisons of h-TENG with different wing deflected angles at a frequency of 2 Hz. **i** Comparisons of output power generation of morphing wings with unidirectional deflection and bidirectional motion at an excitation frequency of 3 Hz and a deflected angle of 20°

Recently, there is a rapid growth of unmanned aerial vehicles (UAVs) in various applications ranging from military security and surveillance to civil infrastructure inspection, mainly due to their advantages such as high mobility, low maintenance cost and ease of deployment, etc. (Fig. 6a). Recent advances in morphing UAVs demonstrate great superiorities compared to those traditional rigid UAVs, such as reduced weight without transmission mechanisms, better aerodynamic performance for hingeless and contoured control surfaces, and multiple missions with single morphing aircraft systems.

One of the critical challenges of morphing UAVs is the small and lightweight fuselage, which restricts the battery size and capacity, and limits the UAVs flight time and range. One promising strategy is to harvest energy from aircraft during flights and convert it into electricity, and the kinetic energy generated from wing vibrations would be an ideal choice. Meanwhile, honeycomb cellular mesostructures have been extensively used in these UAVs since the structures have excellent energy absorption properties and considerable weight to strength ratios. In this work, the lightweight and compact h-TENG skin is fabricated and installed into morphing wings to replace honeycomb cellular mesostructure for converting kinetic energy to electricity (Fig. 6b). This work is the first study to integrate the TENG device into UAV morphing wing energy harvesting application.

Figure 6c shows the fabricated morphing wing integrated with the h-TENG device. The dimensions of flap length and depth of the morphing wing are 300 and 40 mm, respectively. The size and parameters of our UAV are mainly based on the NACA Airfoil Series. Detailed dimensions of the profile are further calculated by the design parameters of NACA 0012 Airfoil. For morphing wing technology, 1 Hz of the wing actuation is often used during cruising. However, during the taking off, rapid wing maneuver and landing period, the aircraft needs a quicker actuating of the morphing wing (usually between 2 and 3 Hz). The motion and deflection of the morphing wing is controlled using a steering engine connected with a crank axle. The angle (*θ*) between the center chord line of the rigid part and the center chord of the tail (flexible wing) is a crucial control factor for calibration. In the experiment, the angles (*θ*) and excited frequencies *(f*) are varied to be *θ* = 10°, 20°, 30° and *f* = 1 Hz, 2 Hz, 3 Hz, respectively. Figures 6d–f show the obtained output voltage waveforms of h-TENG at different excitation frequencies under different deflected angles, respectively. Video S3 shows the dynamic demonstration of h-TENG power generation from the oscillations of the morphing wing. The generated power can continuously lighten up dozens of LEDs. Figure 6g, h show the comparisons of output voltage waveforms and output powers when the morphing wings are deflected into different angles, respectively. It can be seen that the maximum output power of 275 µW is achieved at the deflected angle of 30° and a load resistance of 21 MΩ. Figure 6i shows the comparisons of power generation of morphing wings with unidirectional deflection and bidirectional motions at a continuous excitation of 3 Hz and a deflected angle of 20°, indicating that the output voltage can be further amplified by four times with bidirectional continuous operation.

## Conclusions

In this work, we have demonstrated a hierarchical honeycomb structure-inspired electret/triboelectric nanogenerator for applications in self-powered insole plantar pressure mapping and aircraft morphing wing energy harvesting. The proposed h-TENG has been successfully fabricated by thermoplastic molding and wafer-level bonding with multilayered wavy PET/AgNWs/FEP thin films. The porous honeycomb architecture of h-TENG has divided the large and single hollow space into numerous energy generation cells, increasing the overall capacitance changes even at tiny displacement and amplifying the output performance. A corona discharging process has been further utilized to maximize the thin film electret's surface charge density. Therefore, excellent energy conversion performance has been readily obtained due to the synergized merits of these two strategies. With hand tapping excitations, an instantaneous open-circuit voltage and output power of up to 1207 V and 12.4 mW has been achieved, respectively, corresponding to a remarkable peak power density of 0.275 mW cm^−3^ and 2.48 mW g^−1^.

The unique honeycomb-inspired architecture and elastic material property make the h-TENG device capable of self-rebounding to its original state without any auxiliary resilient supports, making the whole device simple, lightweight and compact. Therefore, the flexible and transparent h-TENG has been further applied to wearable devices for real-time insole plantar pressure mapping and instantaneous tactile imaging. Further investigation has demonstrated its applicability of replacing the honeycomb cellular mesostructure of UAVs for converting flapping wing motions into electricity for the first time. The results of this research have demonstrated the versatility and viability of proposed h-TENG to realize a variety of self-powered and energy harvesting applications.

## Supplementary Information

Below is the link to the electronic supplementary material.

Supplementary file1 (MP4 5108 KB)

Supplementary file2 (MP4 4675 KB)

Supplementary file3 (MP4 2955 KB)

Supplementary file4 (PDF 532 KB)

## References

[1] Wang ZL (2018). Nanogenerators, self-powered systems, blue energy, piezotronics and piezo-phototronics—a recall on the original thoughts for coining these fields. Nano Energy.

[2] Jie Y, Ma J, Chen Y, Cao X, Wang N (2018). Efficient delivery of power generated by a rotating triboelectric nanogenerator by conjunction of wired and wireless transmissions using maxwell's displacement currents. Adv. Energy Mater..

[3] Zhang X-S, Han M, Kim B, Bao J-F, Brugger J (2018). All-in-one self-powered flexible microsystems based on triboelectric nanogenerators. Nano Energy.

[4] Wu Z, Yang X, Wu J (2021). Conductive hydrogel- and organohydrogel-based stretchable sensors. ACS Appl. Mater. Interfaces.

[5] Wen D-L, Liu X, Deng H-T, Sun D-H, Qian H-Y (2019). Printed silk-fibroin-based triboelectric nanogenerators for multi-functional wearable sensing. Nano Energy.

[6] Zhang X, Shan X, Xie T, Miao J (2020). A new sensor inspired by the lateral-line system of fish using the self-powered d33 mode piezoelectric diaphragm for hydrodynamic sensing. Mech. Syst. Signal Proc..

[7] Wen F, Sun Z, He T, Shi Q, Zhu M (2020). Machine learning glove using self-powered conductive superhydrophobic triboelectric textile for gesture recognition in vr/ar applications. Adv. Sci..

[8] Jiang Q, Chen B, Zhang K, Yang Y (2017). Ag nanoparticle-based triboelectric nanogenerator to scavenge wind energy for a self-charging power unit. ACS Appl. Mater. Interfaces.

[9] Jiang Q, Chen B, Yang Y (2018). Wind-driven triboelectric nanogenerators for scavenging biomechanical energy. ACS Appl. Energy Mater..

[10] T. He, X. Guo, C. Lee, Flourishing energy harvesters for future body sensor network: from single to multiple energy sources. iScience **24**(1), 101934 (2021). 10.1016/j.isci.2020.101934 10.1016/j.isci.2020.101934 PMC7773596 33392482

[11] S. Zhukov, H. von Seggern, X. Zhang, Y. Xue, O. Ben Dali et al., Microenergy harvesters based on fluorinated ethylene propylene piezotubes. Adv. Eng. Mater. **22**(5), 1901399 (2020). 10.1002/adem.201901399

[12] Tao K, Yi H, Tang L, Wu J, Wang P (2019). Piezoelectric zno thin films for 2DOF MEMS vibrational energy harvesting. Surf. Coat. Technol..

[13] Yang K, Wang J, Yurchenko D (2019). A double-beam piezo-magneto-elastic wind energy harvester for improving the galloping-based energy harvesting. Appl. Phys. Lett..

[14] Y. Li, Q. Cao, W. Zhang, Y. Zhang, J.A. Cao, A miniaturized electromagnetic energy harvester with volt-level output based on stacked flexible coils. Smart Mater. Struct. **27**(11), 115040 (2018). 10.1088/1361-665X/aae239

[15] Ueno T (2015). Performance of improved magnetostrictive vibrational power generator, simple and high power output for practical applications. J. Appl. Phys..

[16] Zhao L-C, Zou H-X, Yan G, Liu F-R, Tan T (2019). Magnetic coupling and flextensional amplification mechanisms for high-robustness ambient wind energy harvesting. Energy Conv. Manag..

[17] Tan QX, Fan KQ, Tao K, Zhao LY, Cai ML (2020). A two-degree-of-freedom string-driven rotor for efficient energy harvesting from ultra-low frequency excitations. Energy.

[18] Yan B, Ma H, Zheng W, Jian B, Wang K (2019). Nonlinear electromagnetic shunt damping for nonlinear vibration isolators. IEEE/ASME Trans. Mechatron..

[19] Luo A, Zhang Y, Dai X, Wang Y, Xu W (2020). An inertial rotary energy harvester for vibrations at ultra-low frequency with high energy conversion efficiency. Appl. Energy.

[20] Tao K, Wu J, Tang L, Hu L, Lye SW (2017). Enhanced electrostatic vibrational energy harvesting using integrated opposite-charged electrets. J. Micromech. Microeng..

[21] Bi M, Wang S, Wang X, Ye X (2017). Freestanding-electret rotary generator at an average conversion efficiency of 56%: theoretical and experimental studies. Nano Energy.

[22] Z. Yang, L. Tang, K. Tao, K. Aw, A broadband electret-based vibrational energy harvester using soft magneto-sensitive elastomer with asymmetrical frequency response profile. Smart Mater. Struct. **28**(10), 10LT02 (2019). 10.1088/1361-665X/ab3ae1

[23] Guo X, Zhang Y, Fan K, Lee C, Wang F (2020). A comprehensive study of non-linear air damping and “pull-in”effects on the electrostatic energy harvesters. Energy Conv. Manag..

[24] Slabov V, Kopyl S, dos Santos MPS, Kholkin AL (2020). Natural and eco-friendly materials for triboelectric energy harvesting. Nano-Micro Lett..

[25] Zou H, Zhang Y, Guo L, Wang P, He X (2019). Quantifying the triboelectric series. Nat. Commun..

[26] Zhang Y, Zeng QX, Wu Y, Wu J, Yuan SL (2020). An ultra-durable windmill-like hybrid nanogenerator for steady and efficient harvesting of low-speed wind energy. Nano-Micro Lett..

[27] Wang P, Pan L, Wang J, Xu M, Dai G (2018). An ultra-low-friction triboelectric–electromagnetic hybrid nanogenerator for rotation energy harvesting and self-powered wind speed sensor. ACS Nano.

[28] Ji Y, Zhang K, Yang Y (2018). A one-structure-based multieffects coupled nanogenerator for simultaneously scavenging thermal, solar, and mechanical energies. Adv. Sci..

[29] Chen B, Yang Y, Wang ZL (2018). Scavenging wind energy by triboelectric nanogenerators. Adv. Energy Mater..

[30] Zhu M, Yi Z, Yang B, Lee C (2021). Making use of nanoenergy from human – nanogenerator and self-powered sensor enabled sustainable wireless iot sensory systems. Nano Today.

[31] Shan X, Li H, Yang Y, Feng J, Wang Y (2019). Enhancing the performance of an underwater piezoelectric energy harvester based on flow-induced vibration. Energy.

[32] Wu YP, Qiu JH, Zhou SP, Ji HL, Chen Y (2018). A piezoelectric spring pendulum oscillator used for multi-directional and ultra-low frequency vibration energy harvesting. Appl. Energy.

[33] Zhou S, Zuo L (2018). Nonlinear dynamic analysis of asymmetric tristable energy harvesters for enhanced energy harvesting. Commun. Nonlinear Sci..

[34] Ma X, Zhang X, Fang P (2017). Flexible film-transducers based on polypropylene piezoelectrets: Fabrication, properties, and applications in wearable devices. Sens. Actuator A-Phys..

[35] Wang J, Tang L, Zhao L, Hu G, Song R (2020). Equivalent circuit representation of a vortex-induced vibration-based energy harvester using a semi-empirical lumped parameter approach. Int. J. Energy Res..

[36] Zhang K, Wang S, Yang Y (2016). A one-structure-based piezo-tribo-pyro-photoelectric effects coupled nanogenerator for simultaneously scavenging mechanical, thermal, and solar energies. Adv. Energy Mater..

[37] Ji Y, Zhang K, Wang ZL, Yang Y (2019). Piezo–pyro–photoelectric effects induced coupling enhancement of charge quantity in batio3 materials for simultaneously scavenging light and vibration energies. Energy Environ. Sci..

[38] Jin T, Sun Z, Li L, Zhang Q, Zhu M (2020). Triboelectric nanogenerator sensors for soft robotics aiming at digital twin applications. Nat. Commun..

[39] Guo H, Yeh MH, Zi Y, Wen Z, Chen J (2017). Ultralight cut-paper-based self-charging power unit for self-powered portable electronic and medical systems. ACS Nano.

[40] Zhong J, Zhu H, Zhong Q, Dai J, Li W (2015). Self-powered human-interactive transparent nanopaper systems. ACS Nano.

[41] Niu S, Wang X, Yi F, Zhou YS, Wang ZL (2015). A universal self-charging system driven by random biomechanical energy for sustainable operation of mobile electronics. Nat. Commun..

[42] Li S, Wang J, Peng W, Lin L, Zi Y (2017). Sustainable energy source for wearable electronics based on multilayer elastomeric triboelectric nanogenerators. Adv. Energy Mater..

[43] Bai P, Zhu G, Lin ZH, Jing Q, Chen J (2013). Integrated multilayered triboelectric nanogenerator for harvesting biomechanical energy from human motions. ACS Nano.

[44] Wen X, Yang W, Jing Q, Wang ZL (2014). Harvesting broadband kinetic impact energy from mechanical triggering/vibration and water waves. ACS Nano.

[45] Yang W, Chen J, Jing Q, Yang J, Wen X (2014). 3d stack integrated triboelectric nanogenerator for harvesting vibration energy. Adv. Funct. Mater..

[46] Wang J, Wen Z, Zi Y, Zhou P, Lin J (2016). All-plastic-materials based self-charging power system composed of triboelectric nanogenerators and supercapacitors. Adv. Funct. Mater..

[47] Zhou T, Zhang L, Xue F, Tang W, Zhang C (2016). Multilayered electret films based triboelectric nanogenerator. Nano Res..

[48] Gao L, Hu D, Qi M, Gong J, Zhou H (2018). A double-helix-structured triboelectric nanogenerator enhanced with positive charge traps for self-powered temperature sensing and smart-home control systems. Nanoscale.

[49] Zhang LM, Han CB, Jiang T, Zhou T, Li XH (2016). Multilayer wavy-structured robust triboelectric nanogenerator for harvesting water wave energy. Nano Energy.

[50] Wang X, Niu S, Yi F, Yin Y, Hao C (2017). Harvesting ambient vibration energy over a wide frequency range for self-powered electronics. ACS Nano.

[51] Xu M, Wang P, Wang Y-C, Zhang SL, Wang AC (2018). A soft and robust spring based triboelectric nanogenerator for harvesting arbitrary directional vibration energy and self-powered vibration sensing. Adv. Energy Mater..

[52] Chen X, Han M, Chen H, Cheng X, Song Y (2017). A wave-shaped hybrid piezoelectric and triboelectric nanogenerator based on p(VDF-TRFE) nanofibers. Nanoscale.

[53] Feng Y, Zheng Y, Rahman ZU, Wang D, Zhou F (2016). Based triboelectric nanogenerators and their application in self-powered anticorrosion and antifouling. J. Mater. Chem. A.

[54] Guo H, Chen J, Leng Q, Xi Y, Wang M (2015). Spiral-interdigital-electrode-based multifunctional device: Dual-functional triboelectric generator and dual-functional self-powered sensor. Nano Energy.

[55] Cheng X, Song Z, Miao L, Guo H, Su Z (2017). Wide range fabrication of wrinkle patterns for maximizing surface charge density of a triboelectric nanogenerator. Microelectromech. Syst..

[56] Xia X, Chen J, Guo H, Liu G, Wei D (2017). Embedding variable micro-capacitors in polydimethylsiloxane for enhancing output power of triboelectric nanogenerator. Nano Res..

[57] Lai YC, Hsiao YC, Wu HM, Wang ZL (2019). Waterproof fabric-based multifunctional triboelectric nanogenerator for universally harvesting energy from raindrops, wind, and human motions and as self-powered sensors. Adv. Sci..

[58] Wang S, Xie Y, Niu S, Lin L, Liu C (2014). Maximum surface charge density for triboelectric nanogenerators achieved by ionized-air injection: Methodology and theoretical understanding. Adv. Mater..

[59] Xu L, Bu TZ, Yang XD, Zhang C, Wang ZL (2018). Ultrahigh charge density realized by charge pumping at ambient conditions for triboelectric nanogenerators. Nano Energy.

[60] Gong S, Zhang J, Wang C, Ren K, Wang ZL (2019). Monocharged electret nanogenerators: a monocharged electret nanogenerator-based self-powered device for pressure and tactile sensor applications. Adv. Funct. Mater..

[61] Zhang X-S, Han M-D, Wang R-X, Zhu F-Y, Li Z-H (2013). Frequency-multiplication high-output triboelectric nanogenerator for sustainably powering biomedical microsystems. Nano Lett..

[62] Gao T, Zhao K, Liu X, Yang Y (2017). Implanting a solid li-ion battery into a triboelectric nanogenerator for simultaneously scavenging and storing wind energy. Nano Energy.

[63] Liu X, Zhao K, Yang Y (2018). Effective polarization of ferroelectric materials by using a triboelectric nanogenerator to scavenge wind energy. Nano Energy.

[64] Z. Zhang, T. He, M. Zhu, Z. Sun, Q. Shi et al., Deep learning-enabled triboelectric smart socks for iot-based gait analysis and vr applications. NPJ Flexible Electron. **4**(1), 29 (2020). 10.1038/s41528-020-00092-7

[65] Quan T, Wu Y, Yang Y (2015). Hybrid electromagnetic–triboelectric nanogenerator for harvesting vibration energy. Nano Res..

[66] Zhang H, Lu Y, Ghaffarinejad A, Basset P (2018). Progressive contact-separate triboelectric nanogenerator based on conductive polyurethane foam regulated with a bennet doubler conditioning circuit. Nano Energy.

[67] Fang C, Tong T, Bu T, Cao Y, Xu S (2020). Overview of power management for triboelectric nanogenerators. Adv. Intell. Syst..

[68] Cheng X, Tang W, Song Y, Chen H, Zhang H (2019). Power management and effective energy storage of pulsed output from triboelectric nanogenerator. Nano Energy.

[69] Tao K, Yi HP, Yang Y, Chang HL, Wu J (2020). Origami-inspired electret-based triboelectric generator for biomechanical and ocean wave energy harvesting. Nano Energy.

[70] Xiao X, Zhang X, Wang S, Ouyang H, Chen P (2019). Honeycomb structure inspired triboelectric nanogenerator for highly effective vibration energy harvesting and self-powered engine condition monitoring. Adv. Energy Mater..

[71] Feng L, Liu G, Guo H, Tang Q, Pu X (2018). Hybridized nanogenerator based on honeycomb-like three electrodes for efficient ocean wave energy harvesting. Nano Energy.

[72] Xia X, Liu G, Guo H, Leng Q, Hu C (2015). Honeycomb-like three electrodes based triboelectric generator for harvesting energy in full space and as a self-powered vibration alertor. Nano Energy.

[73] Tao K, Tang L, Wu J, Lye SW, Chang H (2018). Investigation of multimodal electret-based mems energy harvester with impact-induced nonlinearity. J. Microelectromech. Syst..

