# Supplementary material for: Hierarchical Honeycomb-Structured Electret/Triboelectric Nanogenerator for Biomechanical and Morphing Wing Energy Harvesting
Source: Nanomicro Lett. 2021 May 10;13:123. doi: 10.1007/s40820-021-00644-0 (PMC8110617; doi:10.1007/s40820-021-00644-0)
Supplement: Supplementary file 4 — Supplementary file4 (PDF 532 KB) [file 40820_2021_644_MOESM4_ESM.pdf]

Supporting Information for

Hierarchical Honeycomb-Structured Electret/Triboelectric Nanogenerator for Biomechanical and Morphing Wing Energy Harvesting

Kai Tao[1], Zhensheng Chen[1], Haiping Yi[1], Ruirong Zhang[1], Qiang Shen[1], Jin Wu[2], Lihua Tang[3], Kangqi Fan[4], Yongqing Fu[5], Jianmin Miao[6,]*, Weizheng Yuan[1,]*

[1]Ministry of Education Key Laboratory of Micro and Nano Systems for Aerospace, Northwestern Polytechnical University, Xi'an 710072, P. R. China

[2]State Key Laboratory of Optoelectronic Materials and Technologies, Key Laboratory of Display Material and Technology, School of Electronics and Information Technology, Sun Yat-sen University, Guangzhou 510275, P. R. China

[3]Department of Mechanical Engineering, University of Auckland, 20 Symonds Street, Auckland 1010, New Zealand

[4]School of Mechano-Electronic Engineering, Xidian University, Xi'an 710071, P. R. China

[5]Faculty of Engineering and Environment, Northumbria University, Newcastle upon Tyne, NE1 8ST, UK

[6]School of Electronic Information and Electrical Engineering, Shanghai Jiao Tong University, Shanghai 200240, P. R. China

*Corresponding authors. E-mail: jmmiao@sjtu.edu.cn (Jianmin Miao); yuanwz@nwpu.edu.cn (Weizheng Yuan)

Supplementary Table and Figures

Table S1 Comparison of the state-of-the-art TENGs with multilayered structures

Refs.	Substrate material	Main structure	Connected method	Volume (cm^3)	Area (cm^2)	Weight (g)	V_{oc} (V)	I_{sc} (μA)	Power density (mW cm^{-3})
[43]	Kapton	Wavy	Bond	12.25	25	-	96	10.85	0.039*
[33]	Silicone	Wavy	Paste	432*	108*	335*	52	16.2	0.009*
[41]	Kapton	Zigzag	Fold	44.46	29.64	3150	480	400	0.102
[35]	Kapton	Wavy	Tape	60*	50	380*	72	32	0.033
[34]	Kapton	Origami	Fold	13.72	13.3	7	215	660	10.24
[32]	Kapton	Zigzag	Fold	47.42	29.64	29.9	700	0.2	0.007*
[31]	PET	Arc shaped	Tape	2*	4	7	28	0.4*	0.004*
[30]	Paper	Kirigami	Assemble	22.4	11.23	8.96	90	15	0.001*
[36]	Acrylic	Stack	Assemble	210	7	230*	303	1140	0.349*
[37]	Acrylic	Stack	Paste	45	9	30.42*	48	8	0.106
[38]	Acrylic	Plane	Paste	3.38*	1.69	2.6*	2450	108	0.118*
[40]	Kapton	Wavy	Assemble	4706	64	510	250	150	0.004
[42]	Silicone	Helical	Fold	54	9	-	85	5	0.004
[39]	Copper	Dual helix	Fold	45	9	10	460	140	0.201
[56]	LCP	Origami	Fold	16.7	6.2	9.3	1000	110	0.67
Curr.	Electret	Honeycomb	Paste	45	15	5	1204	68.5	0.275

*The values are estimated from the reported key characterization parameters in the references.

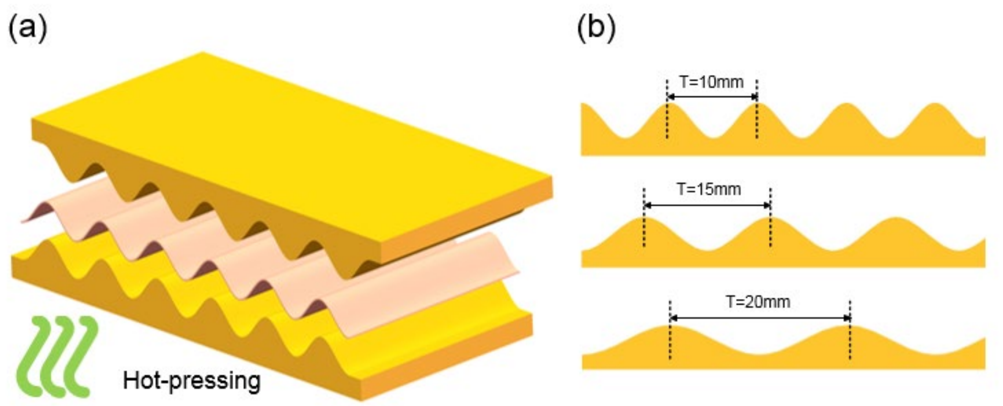

Fig. S1 a Schematic illustration of iron alloy mold for hot-pressing molding process. **b** Schematic illustrations of controlling the period and amplitude of the wavy sheet by adjusting the parameters of iron alloy mold.

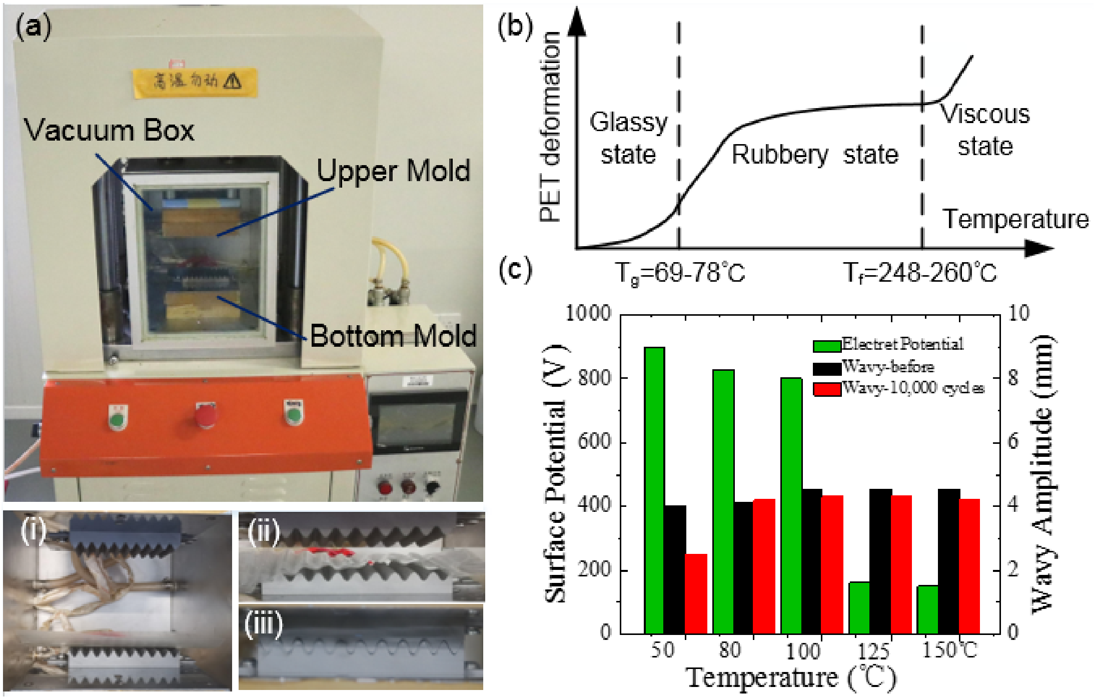

Fig. S2 a Setup of vacuum hot-pressing machine and status of pressing mold: **i** With a large air gap in the initial stage. **ii** Moving close with each other. **iii** Fully pressed with an intimate contact. **b** PET deformation stages under different temperatures: **i** Glassy state at 69 to 78 °C. **ii** Rubbery state. **iii** Viscous state at 248 °C to 260 °C. **c** Stability analysis of electret surface potential and wavy amplitude variations of PET/AgNWs/FEP wavy composites after continuous operation for ~10,000 cycles

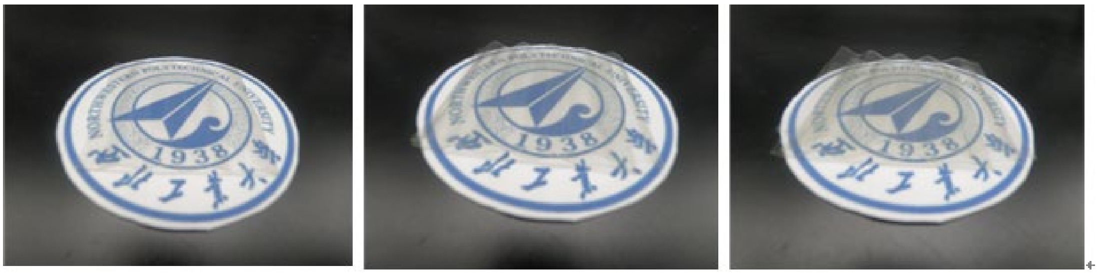

Fig. S3 The photographs of multilayered wavy PET/AgNWs/FEP composites with a good transparency: **a** Single layer. **b** Double layers. **c** Triple layers

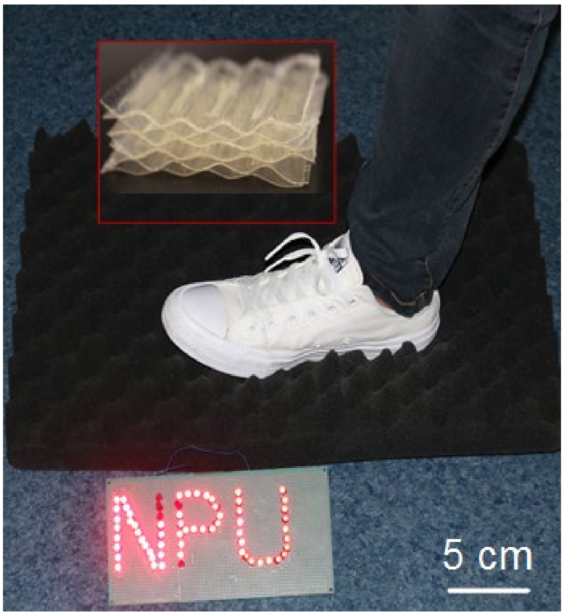

Fig. S4 Photograph of the flexible h-TENG, which can be integrated into a smart foam mat, connected to 70 green LEDs, which can be lightened up simultaneously by the triple-layer h-TENG triggered by footsteps

Video S1 The 75×55 mm^2 LCD can be lightened up for 83 seconds by simply hand pressing the h-TENG device ten times.

Video S2 Real-time insole plantar pressure mapping by integrating an array of h-TENGs in shoes when walking.

Video S3 Dynamic demonstration of h-TENG power generation from the oscillations of the morphing wing. The generated power can continuously lighten up dozens of LEDs.
